# Supplementary material for: Orbitofrontal control of visual cortex gain promotes visual associative learning
Source: Nat Commun. 2020 Jun 3;11:2784. doi: 10.1038/s41467-020-16609-7 (PMC7270099; doi:10.1038/s41467-020-16609-7)
Supplement: Supplementary file 2 — Peer Review File [file 41467_2020_16609_MOESM2_ESM.docx]

Peer Review Information

**Journal:** Nature Communications

**Manuscript title:** Orbitofrontal control of visual cortex gain promotes visual associative learning

**Corresponding author name(s):** Dr Haishan Yao

**Editorial notes:**

**Reviewer comments & decisions:**

**Reviewer comments, first version:**

Reviewers' comments:

Reviewer #1 (Remarks to the Author):

In this paper Liu and colleagues investigate the role of OFC inputs to V1 on go/no-go visual learning. Overall the experiments are technically of a high standard and the analysis well done. There is however a slight disparity between the results and how the authors pitch the story. This is detailed in the comments below. If the authors can correct the pitch of the story to match the results, I would strongly suggest publication.
Suggestions for improvement:

1. The exciting finding of the paper is that interfering with the top-down input from OFC to V1 impairs learning of the go/no-go task. However, the direct effects of stimulating the OFC input to V1 on V1 activity is rather subtle (Figure 1d), and in the Go/No-Go task, only the second phase of the visual response is affected (Figure 4g) by OFC input inhibition. Consistent with this the authors find no acute behavioral effect of stimulating the OFC input on performance of the mouse (Figure 6). Clearly, the acute effects of manipulations of OFC input appear minimal. However, chronic manipulation of OFC input appears to significantly interfere with learning related plasticity in V1 (see also point 2). I would rephrase the manuscript to strengthen and highlight this interpretation. Currently, the manuscript overstates the effects on acute processing (for which the authors find only very little evidence).

2. I would leave this up to the authors as it means substantial addition of experiments, but what one would want to know given the plasticity interpretation proposed above is whether inhibiting the OFC input during learning prevents learning related changes in V1 (Figure 3g,h).

Minor

3. Many of the introductory statements are cumbersome or imprecise. This may be a grammatical issue. E.g. the first sentence of the text (line 19). “The OFC is a critical brain region for using the information about expected outcome…”. Should probably read “The OFC is a brain region critical to an animals’ performance of XXX” or similar. Or on line 25: “Neural signals in the OFC are used by other brain regions” should probably read “Direct output from the OFC to other brain regions is necessary for xyz”. Similarly, for the first sentence of the abstract, etc.

4. Line 52. “wrong trials” should probably read “incorrect trails” and should be introduced. E.g. “in trials in which the animal performed correctly (incorrectly)…”

5. Figure 1a,b (and other similar figures). The resolution is poor in these figures, and they look overexposed. This could be a pdf rendering issue.

6. Figure 4. I would match top and bottom part of the figure in layout.


Reviewer #2 (Remarks to the Author):

The major conclusion of the paper is that OFC projection to V1 impact strongly SST interneurons leading to a reduction of the visual evoked response in V1. This circuit seems to be involved the reduction of V1 neuron response to the non-rewarded No-Go visual stimulus when mice are engaged in a Go/No-Go visual discrimination task. Selective manipulations of OFC-to-V1 projection do affect the response of V1 neurons in an expected maner when the mice are engage in the task but however, does not affect the mice performance. On the other hand, selective inhibition of OFC-to-V1 projection or direct stimulation of SST interneurons in V1 during the learning of the task, significantly impairs or improves the learning, respectively. The role, and mechanisms of action, of top-down inputs to primary sensory areas in the context of learned perceptual decision-making tasks is still poorly understood and certainly of high importance and broad interest for the neuroscience community. The results presented in this study add a new and important piece to this puzzle.

Overall the study is well conducted. The authors have combined many different state-of-the-art methods and the amount and quality of the results is impressive and overall convincing. I do not see any flaws in the data analysis and statistics applied to the results. I definitely believe this paper will be of importance and have a strong impact in the field. Nevertheless, I believe that the paper can be further improved, at least with additional analysis and clarification of some points, to make it suitable for publication in Nature Communications. Additional experiments (though not compulsory) would also help making the paper stronger:

- First, I tend to disagree with one of the main conclusion of the paper, namely that the activity of OFC neurons projecting to V1 mediate the suppression of the response of V1 neuron to the non-rewarded NoGo stimulus. In fact, it seems to me that what the authors show is a disinhibition of V1 neurons in trials during which the animal licked (Hit and FA). Indeed V1-projecting OFC neurons do not show any response to NoGo stimulus in CR trials, but show a prominent inhibition following both Go and No-Go stimuli in Hit and FA trials, respectively (fig. 5f). In fact, the authors should compare the response of OFC neurons between Hit and FA. In good agreement, inhibition of OFC-to-V1 projection during Hit and FA trials have no effect on the response of V1 neurons (see figure 4). Thus, the increase in neuronal response of V1 neurons during the late phase (waiting period) for both Hit trials and FA trials seems to result from a disinhibition of V1 neurons mediated by the decreased firing of V1-projecting OFC neurons. And as such, this is a very interesting finding but it comes with a quite different interpretation as the one proposed by the authors for the role of the OFC.

- The motor activity should be more carefully taken into account in the analysis. From the Method section, I understand is that despite the fact that mice had to wait the end of the waiting period to obtain the reward, mice were not directly discouraged to lick during the stimulus presentation or the waiting period. Even though the mean first lick latency was between 400 and 500 ms, it does not mean that, at population level, licking did not occur within 500 ms in a significant number of trials. In fact, when looking at figure Sup 6j, it seems that the licking rate increases during the waiting periods for both Hit and FA trials with a timing very similar to the late neuronal response in V1. For the analysis of the neuronal activity in V1 or OFC, the authors did not make any selection for trials without early licks (i.e. licking before the 500 ms analyzed). This raises the question as to whether the late increase in neuronal response in V1 (Figure 3f and j) is due to perception/decision or simply reflects increased motor activity. Thus, it would be important to show the evoked response of V1 (and OFC) neurons for selected trials in which no licking occurred within the first 500 ms following stimulus onset. Even better, the authors could select trials without motor activity based on the behavioral video analysis (See Sup Fig. 5).
In line with this idea, the authors make a strong statement that the activity of OFC neurons is not correlated to licking (Sup Fig 6k) whereas the PSTHs for Hit, FA and CR strongly suggest otherwise. The authors should compute lick triggered PSTHs (both for V1 and OFC neurons) to clarify this point.
Also, a finer analysis of the activity of OFC neurons showing cell-by-cell variability would be of great interest (for instance the proportion of neurons with positive, negative or no response).

- The author should distinguish and analyze the early sensory evoked response vs the late evoked response and discuss it in the discussion. The evoked activity of V1 neurons during the task show a clear early sensory response that does not seem to be affected by behavioral outcome and a late response (the one the author have analyzed) that correlates well with the mouse performance (lick / no lick). Interestingly, this pattern of response is very similar to the activity evoked in the primary somatosensory cortex of mice engaged in related Go/NoGo tasks (Sachidhanandam et al., Nature Neuroscience 2013; Kwon et al., Nature Neuroscience 2016). The origin and cellular mechanisms of the late choice-related activity is largely debated and the current study contributes greatly to this understanding. Alternatively, many studies have found that early sensory processing in primary sensory areas can be modulated by learning (see Poort et al., Neuronv2015 or Wilmes and Clopath, Nature Communication 2019 for instance) and/or top-down inputs (see Zhang et al. Science 2014 for instance), so analyzing and discussing both early and late neuronal responses in V1 would be of great interest.

- In my opinion, the design of the optogenetic manipulation experiments is not optimal. When examining the effect of OFC-to-V1 projection inhibition on V1 neuronal response, the inactivation should be applied randomly on every trial type (Go and NoGo) - not alternatively on trial blocks and in different groups of mice for Go and NoGo trials. That would allow a safer comparison of the impact of OFC-to-V1 projection on the activity of V1 neurons and on the animals’ performance. However I do believe that the main results and conclusion of these experiments remain valid.
Similarly, when testing the effect on the learning of the task, the manipulation should have been applied on every trial of a given type (every Go or every No-Go trials) and not only on one half of the trials. Altogether, the authors are not doing themselves a favor by showing odd results whereas the most relevant experiments are in fact convincing. For instance, it is quite surprising that the learning is impaired by the inhibition of OFC-to-V1 projection on only half of the No-Go trials (comparison between Jaws and EGFP in figure 6d), when there is in fact no effect on the immediate performance (figure 6 b)! Similarly, how to interpret the effect on CR (that is NoGo trials) when the laser is only applied during the GO trials in figure 7c !? Again, in figure 7e-f, the stimulation of SST neurons has no immediate effect during each session, but improves the learning of the task (7g).
Therefore, I would suggest to merge the figure 6 and 7. The panels 6b-c and 6e-f bring more confusion than they help and could be moved to supplementary material. The design of the experiment testing the activation of OFC-to-V1 projection during learning is not optimal and the results are also confusing. I would also move panels 7b-f to supplementary material and combine figures 6 d and g, and 7g to make a strong figure with a clear and convincing message.

- Finally, The paper would gain very much if the activity of SST neurons was recorded during the task (and possibly the learning). The study concludes on the interesting idea that OFC neurons projecting to V1 would lead to a decreased late response of V1 neurons in NoGo CR trials due to the excitation of SST neurons. Furthermore, the authors convincingly demonstrate that the inhibition of OFC-to-V1 projections during NoGo trials impairs learning, whereas the stimulation SST neurons during NoGo trials improves learning. However, to be complete the study should also show that SST neurons have a higher activity on NoGo CR trials compared to FA or Hit trials, and ideally that their activity increases during learning. Although I consider this point optional, it would clearly add significantly to the impact of the paper and should be possible using photometry like in figure 2.

Additional comments:

- Overall, when analyzing the neuronal activity from extracellular recordings the author should distinguish (or exclude) putative interneurons (Fast-spiking units or short action potentials) from putative excitatory cells (Regular-spiking units or broad action potentials). Although this distinction is not completely reliable, it is a good approximation for interneurons vs pyramidal cells.

- In figure 2a-d, the authors should be more specific about the population of neurons that have been recorded in brain slices. Which neuron type have been patched (most likely pyramidal neurons?) and from which layer(s)?

- In figure 2f, it is not clear how was determined whether OFC axon stimulation evoked a significant response (EPSC) in a given cell (threshold, statistical test?). Also the authors should have used an extracellular solution containing TTX and 4-AP to prevent polysynaptic responses (see Petreanu et al., Nature 2009). As it is, it is impossible to tell whether SST interneurons received direct excitatory drive from OFC or through indirect local excitation.

- What exactly are the naïve mice in figure 3g-h? Are they mice engaged in the task but that have not yet learned the task or simply mice receiving passively the visual stimuli? The authors should show the neuronal responses for the different stimuli in naïve mice too.

- In figure 3k, what are the black and open bars? What is the p value? Are they neurons with non-significant and significant MI?

- It would seems more meaningful to compute the MI is a similar way than the SI (Lick trial – No Lick trial)/(Lick +No Lick).

Minor:

- figure 4: typo ‘stumulus’ should be ‘stimulus’
- figure 4c: describe what the two graphs are (I suppose mean and single neurons). I do not understand the label at the bottom of the two graphs (abscise).
- sup fig. 6. Graph 6i.
- line 360: ‘… their response to the No-Go stimulus…’
- line 527 : I think this sentence is misleading. I believe that the activation of SST neurons by OFC inputs likely result in the inhibition of PV and possibly VIP neurons, not the other way around?!

Sincerely,

Sylvain Crochet.

**Author rebuttal, first version:**

**Response to Reviewer 1 (Reviewer's comments in *blue* *italic*):**

*In this paper Liu and colleagues investigate the role of OFC inputs to V1 on go/no-go visual learning. Overall the experiments are technically of a high standard and the analysis well done. There is however a slight disparity between the results and how the authors pitch the story. This is detailed in the comments below. If the authors can correct the pitch of the story to match the results, I would strongly suggest publication.
Suggestions for improvement:*

*1. The exciting finding of the paper is that interfering with the top-down input from OFC to V1 impairs learning of the go/no-go task. However, the direct effects of stimulating the OFC input to V1 on V1 activity is rather subtle (Figure 1d), and in the Go/No-Go task, only the second phase of the visual response is affected (Figure 4g) by OFC input inhibition. Consistent with this the authors find no acute behavioral effect of stimulating the OFC input on performance of the mouse (Figure 6). Clearly, the acute effects of manipulations of OFC input appear minimal. However, chronic manipulation of OFC input appears to significantly interfere with learning related plasticity in V1 (see also point 2). I would rephrase the manuscript to strengthen and highlight this interpretation. Currently, the manuscript overstates the effects on acute processing (for which the authors find only very little evidence).*

Indeed, acute manipulation of OFC projection to V1 affected the outcome-expectancy modulation of V1 responses to the No-Go stimulus, without influencing the immediate performance of mice. Chronic manipulation impaired learning but still did not affect immediate performance.

One possible explanation is that during the waiting period, the mice’s decision was already determined by the responses of OFC neurons, which could be seen from the divergence of response curves between FA and CR trials (Fig. 5f and Supplementary Fig. 8o), so that modulation of V1 responses by OFC projection would not affect the mice’s performance in current trial.

A second possibility is that, as the reviewer pointed out, the effect of optogenetic manipulation on the response modulation in V1 is subtle, and the effect may need to be accumulated over multiple sessions to impact behavioral performance. In our experiment, each block consisted of 20 trials, and laser-on and laser-off blocks were interleaved. For mice without optogenetic manipulation (Fig. 3c, d), behavioral performance in interleaved blocks were not significantly different (Fig. 1 for reviewer), indicating that 20 trials of training is not enough to improve performance. Thus, it is likely that chronic manipulation may allow the effect on V1 response modulation to accumulate over multiple sessions to influence learning. Furthermore, the response modification in V1 may in turn contribute to the update of outcome expectation signal in the OFC, potentially contributing to change of performance in future trials.

We have highlighted the effect of chronic manipulation both in the abstract and in the main text, and added the above discussion in the revised manuscript.

**Fig. 1 for reviewer | Comparison of behavioral performance between blocks of trials.**

Black and red curves represent data for odd and even blocks (20 trials/block) in a session, respectively. Left, discriminability in odd and even blocks was not significantly different. *F*_(1, 12)_ = 0.008, *P* = 0.93. Right, Hit rate in odd and even blocks was not significantly different. *F*_(1, 12)_ = 0.14, *P* = 0.71. CR rate in odd and even blocks was not significantly different. *F*_(1, 12)_ = 0.08, *P* = 0.78. The mice in this figure were the same as those in **Fig. 3c, d** in the manuscript. Two-way repeated measures ANOVA. Error bars, mean ± s.e.m.

*2. I would leave this up to the authors as it means substantial addition of experiments, but what one would want to know given the plasticity interpretation proposed above is whether inhibiting the OFC input during learning prevents learning related changes in V1 (Figure 3g,h).*

We have found that acute inhibition of OFC projection to V1 reduced the magnitude of outcome-expectancy response modulation in V1 (Fig. 4e−m and Supplementary Fig. 6). To test whether chronic manipulation of OFC projection to V1 prevents learning related changes in V1, one would need to record from a group of mice in which the inhibitory opsin Jaws is expressed in the OFC and a control group of mice in which control virus is expressed. For both groups of mice, the electrophysiological recordings should be performed after ~10 sessions’ of training, during which laser stimulation of OFC axons in V1 is applied. As both the learning rate of mice (Fig. 3c, d) and the response modulation of V1 neurons (Fig. 3l) exhibited large variability, it is conceivable that we need to record from a large number of mice in both groups to effectively evaluate the effect of chronic manipulation. Therefore, we did not perform this experiment.

Nevertheless, based on the reviewer’s suggestion that chronic manipulation appears to affect learning by interfering with learning-related changes in V1, we conducted an experiment to address a related question: Is behavioral learning rate related to the degree to which OFC top-down projection modulates V1 responses?

For a group of C57BL/6 mice, we injected AAV-Jaws in the OFC, and trained the mice to perform Go/No-Go task for 12 sessions without laser stimulation in V1. After the training, we performed extracellular recordings from V1, with and without laser stimulation during No-Go trials (Fig. 7a). For each mouse, we computed the laser-induced change in V1 firing rate to the No-Go stimulus in CR trials, and used the median of rate change of all neurons (22.9 ± 3.3 neurons/mouse, s.e.m.) to estimate the efficacy of OFC modulation of V1 responses. For each mouse, we also applied a reinforcement learning model^1,2^ to fit the curve of CR rate over the 12 sessions to obtain a learning rate (Fig. 7a, b). We found that the laser-induced rate change of V1 neurons correlated well with the learning rate of the mouse (Fig. 7c). Thus, the data suggest that the individual differences in learning rate may be attributed to the efficacy of OFC top-down projection to modulate V1 responses. We have added the result of this new experiment in the revised manuscript.

*Minor*

*3. Many of the introductory statements are cumbersome or imprecise. This may be a grammatical issue. E.g. the first sentence of the text (line 19). “The OFC is a critical brain region for using the information about expected outcome…”. Should probably read “The OFC is a brain region critical to an animals’ performance of XXX” or similar. Or on line 25: “Neural signals in the OFC are used by other brain regions” should probably read “Direct output from the OFC to other brain regions is necessary for xyz”. Similarly, for the first sentence of the abstract, etc.*

We thank the reviewer for pointing out these grammatical errors. We have revised these sentences accordingly.

*4. Line 52. “wrong trials” should probably read “incorrect trails” and should be introduced. E.g. “in trials in which the animal performed correctly (incorrectly)…”*

We have revised the sentence according to the reviewer’s advice.

*5. Figure 1a,b (and other similar figures). The resolution is poor in these figures, and they look overexposed. This could be a pdf rendering issue.*

In the revised manuscript we have used figures with high resolution, and used figures not overexposed to show labeled neurons in the OFC and the OFC axons in V1.

*6. Figure 4. I would match top and bottom part of the figure in layout.*

As the number of panels was different for different rows, we were able to match some but not all parts of this figure.


**Response to Reviewer 2 (Reviewer's comments in *blue* *italic*):**

*The major conclusion of the paper is that OFC projection to V1 impact strongly SST interneurons leading to a reduction of the visual evoked response in V1. This circuit seems to be involved the reduction of V1 neuron response to the non-rewarded No-Go visual stimulus when mice are engaged in a Go/No-Go visual discrimination task. Selective manipulations of OFC-to-V1 projection do affect the response of V1 neurons in an expected maner when the mice are engage in the task but however, does not affect the mice performance. On the other hand, selective inhibition of OFC-to-V1 projection or direct stimulation of SST interneurons in V1 during the learning of the task, significantly impairs or improves the learning, respectively. The role, and mechanisms of action, of top-down inputs to primary sensory areas in the context of learned perceptual decision-making tasks is still poorly understood and certainly of high importance and broad interest for the neuroscience community. The results presented in this study add a new and important piece to this puzzle.

Overall the study is well conducted. The authors have combined many different state-of-the-art methods and the amount and quality of the results is impressive and overall convincing. I do not see any flaws in the data analysis and statistics applied to the results. I definitely believe this paper will be of importance and have a strong impact in the field. Nevertheless, I believe that the paper can be further improved, at least with additional analysis and clarification of some points, to make it suitable for publication in Nature Communications. Additional experiments (though not compulsory) would also help making the paper stronger:*

We thank the reviewer for the positive and constructive comments.

*- First, I tend to disagree with one of the main conclusion of the paper, namely that the activity of OFC neurons projecting to V1 mediate the suppression of the response of V1 neuron to the non-rewarded NoGo stimulus. In fact, it seems to me that what the authors show is a disinhibition of V1 neurons in trials during which the animal licked (Hit and FA). Indeed V1-projecting OFC neurons do not show any response to NoGo stimulus in CR trials, but show a prominent inhibition following both Go and No-Go stimuli in Hit and FA trials, respectively (fig. 5f). In fact, the authors should compare the response of OFC neurons between Hit and FA. In good agreement, inhibition of OFC-to-V1 projection during Hit and FA trials have no effect on the response of V1 neurons (see figure 4). Thus, the increase in neuronal response of V1 neurons during the late phase (waiting period) for both Hit trials and FA trials seems to result from a disinhibition of V1 neurons mediated by the decreased firing of V1-projecting OFC neurons. And as such, this is a very interesting finding but it comes with a quite different interpretation as the one proposed by the authors for the role of the OFC.*

We thank the reviewer for raising this point. For the No-Go stimulus, the responses of V1-projecting OFC neurons in correct rejection (CR) trials were not different from the baseline response, and those in false alarm (FA) trials exhibited response decrease relative to the baseline (Fig. 5f). In contrast, V1 responses to the No-Go stimulus were lower in CR than in FA trials (Fig. 3k−n). Optogenetic inactivation of OFC top-down projection during No-Go trials reduced the magnitude of response modulation in V1 by increasing the response in CR trials (Fig. 4e−m). In the revised manuscript, we clarified that the OFC projection to V1 mediated the outcome-expectancy modulation of V1 responses, rather than the suppression of V1 responses to the No-Go stimulus.

We agree with the reviewer that the higher responses of V1 neurons in FA and hit trials likely result from a disinhibition mediated by the decreased firing of V1-projecting OFC neurons. On the other hand, since the learning of Go/No-Go visual task manifested as an improvement in CR rate, our purpose was to study the response feature in CR trials to understand the learning.

As we did not record from the same neurons over multiple sessions of training, we assumed that at the beginning of training the responses of OFC (or V1) neurons to the No-Go stimulus were similar to those in FA trials, and with training the responses gradually became more similar to those in CR trials. In trained mice, optogenetic inactivation of OFC top-down projection led to an increase in V1 responses to the No-Go stimulus in CR but not in FA trials (Fig. 4h), suggesting that OFC projection mediates the suppression of V1 responses to the No-Go stimulus in CR trials. Furthermore, we performed an additional experiment to measure SST interneuron activity in behaving mice, and found that the responses of SST interneurons to the No-Go stimulus were higher in CR than in FA trials (Supplementary Fig. 11). This suggests that OFC top-down projection may recruit SST interneurons to suppress V1 responses in CR trials. As the percentage of CR trials increased with training, the percentage of trials that OFC top-down projection could activate local SST interneurons likely increased, increasing the probability that V1 responses to the No-Go stimulus were reduced. Consistent with our speculation that training increases the recruitment of SST interneurons in V1, we found that activation of SST interneurons during No-Go trials facilitated learning (Fig. 6e). Together, our results suggest that OFC top-down projection promotes learning by recruiting local SST interneurons to reduce V1 responses to reward-irrelevant stimulus.

We have clarified the above issues in the revised manuscript.

*- The motor activity should be more carefully taken into account in the analysis. From the Method section, I understand is that despite the fact that mice had to wait the end of the waiting period to obtain the reward, mice were not directly discouraged to lick during the stimulus presentation or the waiting period. Even though the mean first lick latency was between 400 and 500 ms, it does not mean that, at population level, licking did not occur within 500 ms in a significant number of trials. In fact, when looking at figure Sup 6j, it seems that the licking rate increases during the waiting periods for both Hit and FA trials with a timing very similar to the late neuronal response in V1. For the analysis of the neuronal activity in V1 or OFC, the authors did not make any selection for trials without early licks (i.e. licking before the 500 ms analyzed). This raises the question as to whether the late increase in neuronal response in V1 (Figure 3f and j) is due to perception/decision or simply reflects increased motor activity. Thus, it would be important to show the evoked response of V1 (and OFC) neurons for selected trials in which no licking occurred within the first 500 ms following stimulus onset. Even better, the authors could select trials without motor activity based on the behavioral video analysis (See Sup Fig. 5).*

We performed several analyses to address this issue. First, we computed Pearson’s correlation coefficients between spike rate of V1 (or OFC) neurons and lick rate during the waiting period. The distribution of correlation was not significantly different from zero (Supplementary Fig. 5a and Supplementary Fig. 8k). Second, for the lick-triggered spike histogram computed using licks in the waiting period, we did not observe increase in responses after the time of lick (Supplementary Fig. 5b and Supplementary Fig. 8l, m). Third, we analyzed the responses during the waiting period using those trials in which no lick occurred within the first 500 ms following stimulus onset. For these no-lick trials, the responses of V1 neurons to the Go stimulus were significantly larger than those to the No-Go stimulus, and those to the No-Go stimulus remained significantly lower in CR than in FA trials (Supplementary Fig. 5c−f). Similarly, the responses of V1-projecting OFC neurons to the No-Go stimulus remained significantly lower in FA than in CR trials when we used those trials where no lick occurred within the waiting period (Supplementary Fig. 8n). Thus, the response modulation in V1 (or OFC) could not be attributed to licking movement. We have added the above results in the revised manuscript.

*In line with this idea, the authors make a strong statement that the activity of OFC neurons is not correlated to licking (Sup Fig 6k) whereas the PSTHs for Hit, FA and CR strongly suggest otherwise. The authors should compute lick triggered PSTHs (both for V1 and OFC neurons) to clarify this point. Also, a finer analysis of the activity of OFC neurons showing cell-by-cell variability would be of great interest (for instance the proportion of neurons with positive, negative or no response).*

For both V1 neurons and V1-projecting OFC neurons, the histogram of lick-triggered spikes did not show increase in response after the time of lick (Supplementary Fig. 5b and Supplementary Fig. 8l, m). For finer analysis of the activity of V1-projecting OFC neurons, we further divided the neurons into two groups, in which the evoked response in hit trials were lower and higher than the baseline response (RI < 0 vs RI >0), respectively. For either group of OFC neurons, the histogram of lick-triggered spikes in hit trials did not show increase in response after the time of lick (Supplementary Fig. 8m). We have added these results in the revised manuscript.

*- The author should distinguish and analyze the early sensory evoked response vs the late evoked response and discuss it in the discussion. The evoked activity of V1 neurons during the task show a clear early sensory response that does not seem to be affected by behavioral outcome and a late response (the one the author have analyzed) that correlates well with the mouse performance (lick / no lick). Interestingly, this pattern of response is very similar to the activity evoked in the primary somatosensory cortex of mice engaged in related Go/NoGo tasks (Sachidhanandam et al., Nature Neuroscience 2013; Kwon et al., Nature Neuroscience 2016). The origin and cellular mechanisms of the late choice-related activity is largely debated and the current study contributes greatly to this understanding. Alternatively, many studies have found that early sensory processing in primary sensory areas can be modulated by learning (see Poort et al., Neuronv2015 or Wilmes and Clopath, Nature Communication 2019 for instance) and/or top-down inputs (see Zhang et al. Science 2014 for instance), so analyzing and discussing both early and late neuronal responses in V1 would be of great interest.*

We divided the responses during the waiting period into an early component (< 100 ms) and a late component (>100 ms). We found that the selectivity index (SI) for the early response component was not significantly different from zero, and the SI for the late response component was significant (Fig. 3i), indicating that the difference between responses to Go and No-Go stimuli was more prominent in the late component. Similar to the SI, the modulation index (MI) was significantly different from zero for the late but not for the early response component (Fig. 3n). Furthermore, we found that inactivating OFC axons during No-Go trials did not significantly change the firing rates or MI for the early component, but significantly increased firing rates in CR trials and reduced the amplitude of MI for the late component (Fig. 4j−m). Thus, the results suggest that OFC top-down projection contributes to the modulation of late-component responses in V1. We have added the results and discussion of these analyses in the revised manuscript.

*- In my opinion, the design of the optogenetic manipulation experiments is not optimal. When examining the effect of OFC-to-V1 projection inhibition on V1 neuronal response, the inactivation should be applied randomly on every trial type (Go and NoGo) - not alternatively on trial blocks and in different groups of mice for Go and NoGo trials. That would allow a safer comparison of the impact of OFC-to-V1 projection on the activity of V1 neurons and on the animals’ performance. However I do believe that the main results and conclusion of these experiments remain valid.
Similarly, when testing the effect on the learning of the task, the manipulation should have been applied on every trial of a given type (every Go or every No-Go trials) and not only on one half of the trials. Altogether, the authors are not doing themselves a favor by showing odd results whereas the most relevant experiments are in fact convincing. For instance, it is quite surprising that the learning is impaired by the inhibition of OFC-to-V1 projection on only half of the No-Go trials (comparison between Jaws and EGFP in figure 6d), when there is in fact no effect on the immediate performance (figure 6 b)! Similarly, how to interpret the effect on CR (that is NoGo trials) when the laser is only applied during the GO trials in figure 7c !? Again, in figure 7e-f, the stimulation of SST neurons has no immediate effect during each session, but improves the learning of the task (7g).
Therefore, I would suggest to merge the figure 6 and 7. The panels 6b-c and 6e-f bring more confusion than they help and could be moved to supplementary material. The design of the experiment testing the activation of OFC-to-V1 projection during learning is not optimal and the results are also confusing. I would also move panels 7b-f to supplementary material and combine figures 6 d and g, and 7g to make a strong figure with a clear and convincing message.*

We thank the reviewer for many valuable suggestions on the design of optogenetic manipulation experiments, and we will try to improve our experimental design in the future.

We have followed the reviewer’s advice to move the original Fig. 6b−c, e−f and Fig. 7b−f to supplementary figures, and combine the original Fig. 6d, g and Fig. 7g in the revised manuscript.

We found that optogenetic manipulation of OFC top-down projection did not affect immediate performance but affected learning. One possible explanation is that during the waiting period, the mice’s decision was already determined by the responses of the OFC neurons, which could be seen from the divergence of response curves between FA and CR trials (Fig. 5f and Supplementary Fig. 8o), so that modulation of V1 responses by OFC projection would not affect the mice’s performance in current trial. A second possibility is that the effect of optogenetic manipulation (inactivating OFC projection to V1 or activating local SST interneurons) on V1 responses may need to be accumulated over multiple sessions to impact behavioral performance. Furthermore, the response modification in V1 may in turn contribute to the update of outcome expectation signal in the OFC, potentially contributing to change of performance in future trials. We have added the above discussion in the revised manuscript.

The reviewer was also concerned about how to interpret the effect of activating OFC top-down projection on CR rate. We speculated that activation of OFC axons in V1 likely caused antidromic activation of the OFC neurons, and the activity in OFC may serve as a cue to guide the behavior of mice. To avoid confusion, we plotted the overall correct rate, rather than the hit rate and CR rate, for this experiment (Supplementary Fig. 10).

*- Finally, The paper would gain very much if the activity of SST neurons was recorded during the task (and possibly the learning). The study concludes on the interesting idea that OFC neurons projecting to V1 would lead to a decreased late response of V1 neurons in NoGo CR trials due to the excitation of SST neurons. Furthermore, the authors convincingly demonstrate that the inhibition of OFC-to-V1 projections during NoGo trials impairs learning, whereas the stimulation SST neurons during NoGo trials improves learning. However, to be complete the study should also show that SST neurons have a higher activity on NoGo CR trials compared to FA or Hit trials, and ideally that their activity increases during learning. Although I consider this point optional, it would clearly add significantly to the impact of the paper and should be possible using photometry like in figure 2.*

To examine the activity of SST interneurons using fiber photometry, we injected AAV2/9-hSyn-FLEX-GCaMP6s-WPRE in V1 of SST-Cre mice. In mice that had been trained for at least 4 days, the responses to the No-Go stimulus during the waiting period were significantly higher in CR than in FA trials (Supplementary Fig. 11). This response modulation was consistent with that found for the V1-projecting OFC neurons and may be partly attributed to the OFC projection to V1. We have added this new result in the revised manuscript.

*Additional comments:

- Overall, when analyzing the neuronal activity from extracellular recordings the author should distinguish (or exclude) putative interneurons (Fast-spiking units or short action potentials) from putative excitatory cells (Regular-spiking units or broad action potentials). Although this distinction is not completely reliable, it is a good approximation for interneurons vs pyramidal cells.*

Based on the spike waveforms^3,4^, we classified V1 neurons into broad-spiking and narrow-spiking cells, which correspond to putative excitatory and putative inhibitory neurons. We found that the SIs of both cell types were significantly larger than zero, and the MIs of both cell types were significantly smaller than zero (Supplementary Fig. 4). Thus, outcome-expectancy modulation of V1 responses is observed in both cell types. We have added the result of this analysis in the revised manuscript.

*- In figure 2a-d, the authors should be more specific about the population of neurons that have been recorded in brain slices. Which neuron type have been patched (most likely pyramidal neurons?) and from which layer(s)?*

For slice recordings, the neurons were recorded from layer 2/3. For Fig. 2b, *n =* 7, 12, 1, 1 and 9 for pyramidal, SST, PV, VIP and unidentified neurons, respectively. We have added this information in the revised manuscript. For Fig. 2c, d, the V1 slices were from C57BL/6 mice and we did not identify cell type.

*- In figure 2f, it is not clear how was determined whether OFC axon stimulation evoked a significant response (EPSC) in a given cell (threshold, statistical test?). Also the authors should have used an extracellular solution containing TTX and 4-AP to prevent polysynaptic responses (see Petreanu et al., Nature 2009). As it is, it is impossible to tell whether SST interneurons received direct excitatory drive from OFC or through indirect local excitation.*

To determine whether the laser stimulation-evoked EPSC is significant, we computed the baseline by averaging the responses within 10 ms before laser onset, and compared the peak amplitude of EPSC with the baseline using Wilcoxon signed rank test. In our slice recording experiment, unfortunately we did not use an extracellular solution containing TTX and 4-AP. As the latencies of EPSCs were short (Fig. 2f), it is likely that the SST interneurons receive direct excitatory drive from the OFC.

*- What exactly are the naïve mice in figure 3g-h? Are they mice engaged in the task but that have not yet learned the task or simply mice receiving passively the visual stimuli? The authors should show the neuronal responses for the different stimuli in naïve mice too.*

We have clarified that the naïve mice were those untrained mice passively viewing the stimulus. We have added a figure showing the responses of V1 neurons to different stimuli in untrained mice (Fig. 3g). When we analyzed the PSTHs of neurons in untrained mice, we found that in our original Matlab script the neurons in untrained mice were not screened by the same criterion as those in the trained mice. We have corrected this problem and updated the result for untrained mice in the revised manuscript.

*- In figure 3k, what are the black and open bars? What is the p value? Are they neurons with non-significant and significant MI?*

For each V1 neuron, we tested the statistical significance of MI by comparing the waiting-period responses to No-Go stimulus between CR and FA trials using Wilcoxon rank sum test. The *P* values of this test were used to determine whether the cells were plotted with open (*P* > 0.05) or black bars (*P* < 0.05) in Fig. 3l (original Fig. 3k). This has been clarified in the figure legend and the method.

*- It would seems more meaningful to compute the MI is a similar way than the SI (Lick trial – No Lick trial)/(Lick +No Lick).*

In our study, the learning of Go/No-Go visual task manifested as an improvement of CR rate over training sessions. Because we want to emphasize that the responses of V1 neurons to the No-Go stimulus were lower in CR than in FA trials, we prefer to compute MI as (R_CR_ - R_FA_)/(R_CR_ + R_FA_).

*Minor:

- figure 4: typo ‘stumulus’ should be ‘stimulus’*

We have corrected this figure.

*- figure 4c: describe what the two graphs are (I suppose mean and single neurons). I do not understand the label at the bottom of the two graphs (abscise).*

Fig. 4c was the laser-induced rate change for the responses to the Go stimulus. We have replotted this figure as the distribution of rate change.

*- sup fig. 6. Graph 6i.*

We have corrected this figure in the revised manuscript.

*- line 360: ‘… their response to the No-Go stimulus…’*

We have corrected the sentence in the revised manuscript.

*- line 527 : I think this sentence is misleading. I believe that the activation of SST neurons by OFC inputs likely result in the inhibition of PV and possibly VIP neurons, not the other way around?!*

We have revised this sentence.

**References**

1	Pearce, J. M. & Hall, G. A model for Pavlovian learning: variations in the effectiveness of conditioned but not of unconditioned stimuli. *Psychol. Rev.* **87**, 532-552 (1980).

2	Sutton, R. S. & Barto, A. G. *Reinforcement learning: an introduction.* (Cambridge MA:MIT., 1998).

3	Bakhurin, K. I. *et al.* Differential Encoding of Time by Prefrontal and Striatal Network Dynamics. *J. Neurosci.* **37**, 854-870 (2017).

4	Zhu, Y., Qiao, W., Liu, K., Zhong, H. & Yao, H. Control of response reliability by parvalbumin-expressing interneurons in visual cortex. *Nat. Commun.* **6**, 6802 (2015).

**Reviewer comments, second version:**

REVIEWERS' COMMENTS:

Reviewer #1 (Remarks to the Author):

The authors have somewhat improved the phrasing of the manuscript - but only partially. The title still suggests that OFC exerts a gain control over V1, which I presume technically is true, but rather misleading as the acute effects the authors describe are minimal. This theme the authors follow throughout the rest of the manuscript.

Most importantly, figure 7c and related analysis the authors have now added – in stark contrast to the rest of the work – is simply nonsense in its current form. Please excuse the blunt language, here and in the following, but it is paramount that the authors remove this. The experiment is probably about two orders of magnitude short in sample size to be able to reasonably address the question the authors are interested in. The authors claim to be able to relate the strength of the OFC-V1 projection to the learning rate, animal by animal. The crux lies in measuring the strength of the OFC-V1 projection. The authors do this by injecting an AAV expressing an optogenetic inhibitor in OFC and then proceed to record the stimulation effect from an average of 22 neurons in V1 of each mouse. There are three factors that contribute to variability of the quantification of the “strength of the OFC-V1 projection” here.

1. The bolus size typically varies, animal to animal (i.e. the number of neurons expressing JAWs), by about a factor of 10 (I should add, this is not based on proper quantification, but an estimate based on having done thousands of these AAV injections over the past decade or so).
2. Small variations in the injection location, or animal to animal differences in the exact coordinates of OFC probably contribute another factor of 10 variability in the number of OFC-V1 axons labelled.
3. The variability in response to OFC stimulation is again about a factor of 10.

Combining the large technical variability in expression pattern and level, with the almost comically small number of neurons recorded in V1, makes this measurement underpowered by at least two orders of magnitude. The mere fact that the authors have chosen to include this makes me seriously question the authors understanding of the methods they use. Either the authors remove this or do it properly. The latter would require a group size of somewhere on the order of hundreds of animals and imaging a reasonable fraction of the total population of local V1 neurons, and would likely go far beyond the amount of effort the authors aim to invest in this study.


Reviewer #2 (Remarks to the Author):

In this revised version of their paper, the authors have made serious revisions, carefully and thoroughly addressing the reviewers’ comments. Overall, the authors have addressed all of my concerns and I find the paper now suitable for publication in Nature Communications.

I only have a few minor points that should be corrected before publication but would not require any further reviewing:

- The description of the response of OFC neurons during the task might be confusing sometime. What the authors found is that there is no response of V1 projecting OFC neurons in CR trials, but that there is a significant decrease in firing rate following stimulus onset in Hit and FA trials.
In lines 232-234, I would not state that the ‘responses were reduced’ in some trials and ‘unchanged’ in others but instead that the ‘activity’ or ‘firing rate’ was reduced or unchanged.
In lines 249-251, ‘Given that activating OFC axons in V1 caused response suppression of V1 neurons, the higher responses of V1-projecting OFC neurons in CR than in FA trials may provide an explanation of the observation that V1 responses to the No-Go stimulus were lower in CR trials.’
should be rephrase as :
‘ Given that activating OFC axons in V1 caused response suppression of V1 neurons, the decreased activity of V1-projecting OFC neurons in FA, but not CR, trials may provide an explanation of the observation that V1 responses to the No-Go stimulus were higher in FA trials.’

- In figure 3g the labels for the y-axis are shifted upward. Ideally, the authors should use the same range for the y-axis in panels f and g (possibly 0-0.7).

- In supplementary figure 11: The authors should display the response of SST neurons for longer timescale, like -0.5 to 1s or even longer -1 to 2 s. It would also be very informative to see the response in Hit trials.

- Could the authors briefly describe in the Methods section how they normalized the PTSHs for the evoked responses from extracellular recordings (Fig. 3f,g and k; 4b and g; 5f …)? Do they apply any baseline subtraction?

Sylvain Crochet

**Author rebuttal, second version:**

**Response to Reviewer 1 (Reviewer's comments in *blue* *italic*):**

*The authors have somewhat improved the phrasing of the manuscript - but only partially. The title still suggests that OFC exerts a gain control over V1, which I presume technically is true, but rather misleading as the acute effects the authors describe are minimal. This theme the authors follow throughout the rest of the manuscript.

Most importantly, figure 7c and related analysis the authors have now added – in stark contrast to the rest of the work – is simply nonsense in its current form. Please excuse the blunt language, here and in the following, but it is paramount that the authors remove this. The experiment is probably about two orders of magnitude short in sample size to be able to reasonably address the question the authors are interested in. The authors claim to be able to relate the strength of the OFC-V1 projection to the learning rate, animal by animal. The crux lies in measuring the strength of the OFC-V1 projection. The authors do this by injecting an AAV expressing an optogenetic inhibitor in OFC and then proceed to record the stimulation effect from an average of 22 neurons in V1 of each mouse. There are three factors that contribute to variability of the quantification of the “strength of the OFC-V1 projection” here.

1. The bolus size typically varies, animal to animal (i.e. the number of neurons expressing JAWs), by about a factor of 10 (I should add, this is not based on proper quantification, but an estimate based on having done thousands of these AAV injections over the past decade or so).
2. Small variations in the injection location, or animal to animal differences in the exact coordinates of OFC probably contribute another factor of 10 variability in the number of OFC-V1 axons labelled.
3. The variability in response to OFC stimulation is again about a factor of 10.

Combining the large technical variability in expression pattern and level, with the almost comically small number of neurons recorded in V1, makes this measurement underpowered by at least two orders of magnitude. The mere fact that the authors have chosen to include this makes me seriously question the authors understanding of the methods they use. Either the authors remove this or do it properly. The latter would require a group size of somewhere on the order of hundreds of animals and imaging a reasonable fraction of the total population of local V1 neurons, and would likely go far beyond the amount of effort the authors aim to invest in this study.*

We thank the reviewer for raising this point. We have removed Figure 7 from the revised manuscript.

**Response to Reviewer 2 (Reviewer's comments in *blue* *italic*):**

*In this revised version of their paper, the authors have made serious revisions, carefully and thoroughly addressing the reviewers’ comments. Overall, the authors have addressed all of my concerns and I find the paper now suitable for publication in Nature Communications.*

We thank the reviewer for the constructive suggestions that have helped us to improve the manuscript.

*I only have a few minor points that should be corrected before publication but would not require any further reviewing:

- The description of the response of OFC neurons during the task might be confusing sometime. What the authors found is that there is no response of V1 projecting OFC neurons in CR trials, but that there is a significant decrease in firing rate following stimulus onset in Hit and FA trials.
In lines 232-234, I would not state that the ‘responses were reduced’ in some trials and ‘unchanged’ in others but instead that the ‘activity’ or ‘firing rate’ was reduced or unchanged.*

We have revised the sentence according to the reviewer’s advice.

*In lines 249-251, ‘Given that activating OFC axons in V1 caused response suppression of V1 neurons, the higher responses of V1-projecting OFC neurons in CR than in FA trials may provide an explanation of the observation that V1 responses to the No-Go stimulus were lower in CR trials.’
should be rephrase as :
‘ Given that activating OFC axons in V1 caused response suppression of V1 neurons, the decreased activity of V1-projecting OFC neurons in FA, but not CR, trials may provide an explanation of the observation that V1 responses to the No-Go stimulus were higher in FA trials.’*

We have revised the sentence according to the reviewer’s advice.

*- In figure 3g the labels for the y-axis are shifted upward. Ideally, the authors should use the same range for the y-axis in panels f and g (possibly 0-0.7).*

We have revised Figure 3f and 3g according to the reviewer’s advice.

*- In supplementary figure 11: The authors should display the response of SST neurons for longer timescale, like -0.5 to 1s or even longer -1 to 2 s. It would also be very informative to see the response in Hit trials.*

For Supplementary Figure 11, we have displayed the response of SST neurons for a longer timescale (-0.5 to 1 s), and added the response in Hit trials.

*- Could the authors briefly describe in the Methods section how they normalized the PTSHs for the evoked responses from extracellular recordings (Fig. 3f,g and k; 4b and g; 5f …)? Do they apply any baseline subtraction?*

In the Methods section, we added the method of normalizing the PSTHs as below:

To obtain the normalized peri-stimulus time histogram (PSTH) over the population of neurons, the firing rates of each neuron were normalized by the maximum of the peak values of PSTHs in the Go and No-Go trials (or in hit, FA and CR trials), and were averaged across neurons.

We did not apply any baseline subtraction

**Reviewer comments, third version:**

**Author rebuttal, third version:**
